# Platforms for Production of Protein-Based Vaccines: From Classical to Next-Generation Strategies

**DOI:** 10.3390/biom11081072

**Published:** 2021-07-21

**Authors:** Raquel Cid, Jorge Bolívar

**Affiliations:** 1 ADL Bionatur Solutions S.A., Av. del Desarrollo Tecnológico 11, 11591 Jerez de la Frontera, Spain; 2 Department of Biomedicine, Biotechnology and Public Health-Biochemistry and Molecular Biology, Campus Universitario de Puerto Real, University of Cadiz, 11510 Puerto Real, Spain

**Keywords:** vaccine, protein expression system, alternative platform, subunit, recombinant antigen

## Abstract

To date, vaccination has become one of the most effective strategies to control and reduce infectious diseases, preventing millions of deaths worldwide. The earliest vaccines were developed as live-attenuated or inactivated pathogens, and, although they still represent the most extended human vaccine types, they also face some issues, such as the potential to revert to a pathogenic form of live-attenuated formulations or the weaker immune response associated with inactivated vaccines. Advances in genetic engineering have enabled improvements in vaccine design and strategies, such as recombinant subunit vaccines, have emerged, expanding the number of diseases that can be prevented. Moreover, antigen display systems such as VLPs or those designed by nanotechnology have improved the efficacy of subunit vaccines. Platforms for the production of recombinant vaccines have also evolved from the first hosts, *Escherichia coli* and *Saccharomyces cerevisiae*, to insect or mammalian cells. Traditional bacterial and yeast systems have been improved by engineering and new systems based on plants or insect larvae have emerged as alternative, low-cost platforms. Vaccine development is still time-consuming and costly, and alternative systems that can offer cost-effective and faster processes are demanding to address infectious diseases that still do not have a treatment and to face possible future pandemics.

## 1. Historical Perspective of Vaccine Development

Although attempts to prevent small-pox infection by variolation was reported in the 15th century in China [1], it was not until 1796 that Edward Jenner identified that inoculation of humans with cowpox virus was protective against subsequent smallpox infection, leading to the development of the first world vaccine [2]. The term *vaccine* was thus originated after the Latin word *vacca*, which means cow [3]. This finding opened the era of vaccination and led to the discovery of further vaccines against other infectious diseases. In 1879 Louis Pasteur established the concept of attenuated microorganisms while studying chicken cholera *Pasteurella multocida* [4] and in 1885, based on the discovery of the infectious agent of rabies by Pierre Galtier he also developed a vaccine for humans using an attenuated strain [5]. The finding of attenuated microorganisms defined the beginning of the first golden age of vaccinology (from Pasteur’s era to 1938), leading to the development of other vaccines, such as live-attenuated (tuberculosis and yellow fever), inactivated (typhoid, cholera, plague and pertussis) and subunit vaccines (tetanus and diphtheria) [6]. Influenza vaccines were developed in the mid-1930s, after identifying that influenza virus could be grown in embryonated eggs, implementing a method that is still used today to manufacture most of influenza vaccines [7]. One of the main limitations in the development of vaccines was the lack of techniques to culture infectious agents, such as viruses, that need host tissue to grow [8]. Advances in culture techniques defined a landmark success in the second golden age of vaccinology (1940–1970), leading to the development of vaccines for viral infections such as poliomyelitis, measles, mumps and rubella [6]. In the early 1970s, advancements in molecular biology led to the development of recombinant DNA technology [9], and over the next 50 years, recombinant expression systems such as *Escherichia coli* (*E. coli*), *Saccharomyces cerevisiae* (*S. cerevisiae*) or baculovirus–insect cells were stablished for foreign protein production, leading to the third phase of vaccine development and the emergence of subunit vaccines [8]. The first subunit vaccine against hepatitis B virus (HBV) was approved in 1986 and was based on the viral surface protein that self-assembled into VLPs using *S. cerevisiae* as expression system [10]. Vaccine design focused on the use of specific antigens from infectious agents, including those that were unable to culture or were highly pathogenic, and recombinant vaccines such as human papillomavirus (HPV) were also developed. Over the last years, advances in genetic engineering have enabled the development of new platforms for protein expression, such as mammalian cells, plants or insect larvae, and also the emergence of new technologies applied to vaccine development, such as nanotechnology.

Nowadays, there are vaccines available to protect against more than 20 human diseases, and the World Health Organization (WHO) estimates that vaccines save between 4 million and 5 million lives every year [11]. Although the market value of veterinary vaccines is modest when compared to human counterparts, veterinary vaccination has also seen many significant advances in technologies over the last years. What is more, many new vaccine technologies may find their first commercial application in veterinary market, and this is reason why veterinary vaccines are more varied. In addition to traditional vaccines based on whole-pathogens and subunit vaccines, new vaccines technologies, such as live genetically modified pathogens, vectored vaccines (that use viruses as delivery systems for foreign antigens) and DNA vaccines, are already approved for their commercialization [12].

The One Health approach has emerged in the recent decades as a new perspective within vaccination strategies. It is a transdisciplinary approach that recognizes the importance of the interconnection between people, animals, plants and their shared environment. One Health issues include several topics related with health threats such as zoonotic diseases, antimicrobial resistance and food safety, among others. Regarding vaccination, zoonotic diseases are of great importance, because they can spread between animals and people, as is the case of Ebola, rabies or West Nile virus. For example, the One Health approach for Ebola aims for a shared benefit, i.e., the vaccination of wild apes in order to protect both apes and human [13].

## 2. Key Aspects in Vaccine Design

### 2.1. The Immune System and the Rationale of Vaccination

Infectious-disease vaccines are based on the controlled exposure to an infectious agent, either whole-cell or specific antigen, with the aim to induce an immune response. The immune system is composed of two different arms, the innate immune system that includes a diverse array of cell types that interact with foreign molecules in a nonspecific manner and the adaptive immune system that, on the contrary, interacts in a specific manner through humoral and cellular responses [8]. The adaptive immune response depends on the interaction of three key cell types: antigen-presenting cells (APCs), thymus-derived lymphocytes (T cells) and bone-marrow-derived lymphocytes (B cells) [3]. Cellular immune response is based on the action of T cells, while humoral immune response involves the activity of antibodies generated by B cells. APCs are specialized in the capture of antigens, subsequent processing and presentation on their surface bounded to a Major Histocompatibility Complex (MHC) I or II for T-cells recognition. B-cell receptors may directly recognize foreign antigens, leading to the production of subclasses of antibodies [3]. Nonetheless, T cells are unable to directly interact with antigens and the mechanism of MHC presentation in addition to appropriate signaling plays a key role in cell activation and is of particular importance in vaccine development in which a T-cell-mediated response is required [14]. T cells are composed by cytotoxic T cells (CTLs) that mediate lysis of autologous cells infected by intracellular pathogens and T helper cells (Th) that are able to stimulate cells of both the innate and adaptive immune system through the secretion of cytokines [8] and also by cell-to-cell interactions (e.g., CD40/CD40L). Based on the profile of the secreted cytokines the responses are classed as T helper 1 (Th1) or T helper 2 (Th2). Th1 response stimulates a cellular based immune response, while Th2 response leads to a humoral immune response [8]. Vaccines are highly dependent on effective adaptive immune responses, and although traditionally strategies for vaccines production have focused on the induction of strong humoral response, currently, a vaccine candidate that has a balanced Th1/Th2 response is considered optimal [15].

Subunit vaccines have advantages over whole-organism vaccines regarding safety and production but in general they tend be less immunostimulatory [16]. This is possibly caused by a reduced ability to crosslink B-cell receptors, as well as a reduced ability to stimulate APCs [17]. Many approaches have been explored to develop more effective subunit vaccines, including the use of adjuvants to stimulate the immune system or the use of nanotechnology to design and generate effective antigen display systems.

### 2.2. Route of Administration

The route of administration is an important issue for the achievement of an adequate protection by vaccination. Most of available vaccines are based on the injectable route of administration, either intramuscular or subcutaneous, that is known to elicit a robust systemic humoral response but confers a weaker T-cell-mediated immunity and mucosal protection [18,19]. In addition to limitations related to immunity, injectable vaccines also require trained personnel for administration and can cause inconveniences to patients. Vaccine methods delivered to the mucosal interface or intradermally are being explored as attractive alternative to needle-based delivery systems, as they are painless and can be self-administered.

Given that a majority of pathogens invade via mucosal surfaces, delivery of vaccines across mucosal surfaces is of great interest to provide immunity at early stages of infection before pathogens are able to cause a widespread infection [20]. A wide range of mucosal vaccination routes are being explored, with oral and intranasal being the most common. Others include ocular, intravaginal and intrarectal [20]. They are also promising candidates to improve overall immunogenicity as vaccination at mucosal surfaces can induce mucosal antibodies (IgA) and cell-mediated immune responses, while still producing a systemic antibody response (IgG) [21,22,23,24]. Current marketed oral vaccines are based on attenuated pathogens [25], still bearing the potential to revert to pathogenic forms [26] and new approaches for the development of safer oral vaccines are demanded. The use of food-grade organisms can offer several advantages for oral vaccines as they can be used at the same time for antigen expression and oral delivery, avoiding costly purification processes [27] and providing stability when orally administered. The development of efficient oral vaccines involves several key aspects, such as the viability/bioactivity when stored at ambient temperature, the characterization of the adjuvant activity of the expression hosts and the balance between immunogenicity and tolerance [27]. The organisms currently used for this purpose are bacteria (*Lactobacillus* and *Bacillus*), yeasts, insects, plants and algae [27].

The only licensed intranasal vaccine for use in humans is for influenza (FluMist^®^ Quadrivalent; AstraZeneca, Cambridge, UK) and consists of a live attenuated form of the virus. The nasal cavity is highly vascularized with a large surface area for antigen uptake; however, most antigens have very little affinity for the nasal epithelium, leading to a fast clearance [28]. To solve this issue, researchers are focusing on improving nasal delivery methods [29].

Another vaccine delivery route capable of triggering both systemic and mucosal immunities is the intradermal route. The skin is composed of a large number of immune cells conforming a great target location for vaccine administration [30]. Recent advancements have led to the development of techniques and instruments that can overcome the difficulties associated with intradermal administration [31]. Among them, solid microneedles, particle injectors and self-administrable patches with coated microprojections or biodegradable needles have been developed [32]. Intradermal vaccination has also been of interest for its ability to elicit equivalent antibody responses at lower doses, a phenomenon typically described as dose sparing [33]. Lowering a vaccine dose could be of great importance for pandemics as huge quantities of doses are required. In this regard, the US Food and Drug Administration (FDA) approved the trivalent inactivated intradermal influenza vaccine for use during the 2012–2013 season, and a quadrivalent formulation was subsequently approved in 2014 [34].

## 3. New Strategies for Vaccine Development

Classical vaccines of whole-pathogen antigens have been irrefutably successful in the control of many of the important veterinary and human infectious diseases but they also possess some limitations and associated potential problems. Although live-attenuated vaccines can induce a robust immune response, they bear the risk of retaining virulence, [35] and although inactivated vaccines are a safer alternative, they usually produce less immunity and need multiple boosters [36,37]. In addition, the production of these vaccines usually requires high-level biosafety facilities implying high production costs. Modern vaccine technologies can overcome these manufacturing issues and generate safer vaccines rapidly and at a low cost. Different strategies for vaccines development include the use of viruses, viral vectors, nucleic acids, proteins or cells [38,39].

Approved vaccines for human use mostly fall into the virus-based and the recombinant-protein-based categories. Among all approaches to develop a vaccine candidate against the emerging coronavirus disease 19 (COVID-19) pandemic, two new types of platforms, mRNA-based and viral-vector-based vaccines have been the first to reach the market due to the rapid advancement in their methodologies [40]. Veterinary vaccines are more varied and along with traditional virus-based and recombinant-protein-based vaccines, live genetically modified, viral vector and DNA vaccines are already in the market [14]. New technology vaccines can also be used as a valuable tool in disease control and eradication programs in animal health by enabling the immunological differentiation between infected and vaccinated animals. These vaccines termed as marker or DIVA (differentiating infected from vaccinated animals) are usually based on recombinant deletion mutants of wild-type pathogens or subunit vaccines [38] and are of great interest in disease control, because they allow us to use serological surveillance for the presence of infection after vaccination. Infection and vaccination can be detected by the use of companion diagnostic tests such as enzyme linked immunosorbent assays (ELISA). DIVA vaccines have been widely used for the control of pseudorabies and classical swine fever (CSF) in pigs and infectious bovine rhinotracheitis in cattle [41].

### 3.1. Pathogen-Based Vaccines

These vaccines consist of the pathogen that causes the infectious disease either in their whole form (as live-attenuated or inactivated pathogens) or as derived split-products (subunit vaccines). Pathogens used as vaccine candidates are viruses, bacteria or parasites, with viruses and bacteria being the predominant types in current marketed vaccines. Live-attenuated and inactivated formulations are still the most widely used to date in human health [38]. The advantages of these methods include well-developed methodologies, well-established regulation policies and existing manufacturing facilities [39]. On the contrary, production of large volumes of pathogens is required, and it demands specialized containment facilities and poses considerable risk to the operators and environment due to the infectious nature of the material [42,43], involving high costs of manufacturing. In addition, manufacturing process is lengthy with large periods until vaccine delivery [44]. Live-attenuated vaccines most closely mimic natural infections, eliciting strong cellular and antibody responses [8], but they also pose risks of uncontrolled replication and have the potential to revert to a pathogenic form [45]. Advancements in genetic engineering have reduced the unpredictability of experimental attenuation and improved the safety associated with live-attenuated pathogens [46]. Killed vaccines consist of the disease-causing pathogen inactivated by chemicals, heat or radiation [45]. They cannot replicate and are safer than live vaccines, but they usually prompt a weaker immune response, and, generally, additional doses or booster shots are required to maintain protection [47,48]. In addition to whole-pathogen vaccines, split-products or subunit vaccines can also be generated from natural sources; however, they tend to reduce immunogenicity when compared with whole formulations [38]. Split-products can be composed of the infectious agent disassembled into its component parts or only on selected components, also known as subunit vaccines. They can be divided into four main categories: protein-based, polysaccharides, conjugates and toxoids [45]. Protein-based subunit vaccines use specific isolated proteins as antigens and in addition to purification from natural sources, they can be produced as heterologous proteins in recombinant systems. As a key aspect of this review, recombinant-protein-based vaccines and the platforms for protein expression will be further detailed within this work. Bacterial subunit vaccines can be divided into toxoid vaccines (detoxified toxins) and capsular polysaccharides vaccines. A variation of them are conjugate vaccines, consisting of an antigen (often the bacterial polysaccharides) covalently attached to a carrier protein (e.g., tetanus or diphtheria toxoid), to improve the immunogenicity of the antigen [49].

Virus-derived lipids (virosomes) are also of great interest for vaccine development. The envelope fraction containing lipids and membrane-associated viral proteins is purified from enveloped viruses and is assembled in vitro for presenting antigens from different pathogens that can be either displayed on the virosome surface or incorporated into its lumen [50]. While virosomes containing envelope proteins from a wide range of viruses have been generated, only virosomes incorporating hemagglutinin (HA) and neuraminidase proteins from the influenza viral envelope have been licensed as human vaccines. Two different virosomes were approved for human health, Inflexal^®^, an influenza vaccine, [51] and Epaxal^®^, a formalin inactivated hepatitis A that is adsorbed to an influenza virosome [52]; however, in 2011, Crucell decided to discontinue these vaccines from the market [53]. Bacterial outer membrane vesicle (OMVs) have also attracted the attention as vaccine platform against bacterial pathogens. OMVs are naturally released from Gram-negative bacteria and are composed of the outer membrane that contains key antigenic components required to elicit a protective immune response. Genetically manipulated bacteria to increase OMV are known as GMMA (Generalized Modules for Membrane Antigens) and can be further manipulated for overexpression of homologous or heterologous protein antigens, which can serve as vaccine antigens [54]. This system have allowed the development of Meningococcal group B Vaccines for human (Bexsero^®^; GSK, Londres, UK), based on the expression of *Neisseria meningitidis* cell surface antigens in *E. coli* in combination with outer membrane vesicles (OMV) from the bacterial membrane of *Neisseria meningitidis* serogroup B.

### 3.2. Viral-Vector-Based Vaccines

Viral vector vaccines work by cloning the antigen of interest into a heterologous virus that will serve as a vector to deliver the foreign gene inside the host cells, leading to its expression [55]. They often provide long-lived immunity, including humoral antibodies, secretory antibodies and cytotoxic T cells [38]. Adenoviruses are the most extended for human vaccines because they are infectious to various cell types, and they show efficient transgene expression, high in vitro growth, lack of genome integration and genetic stability [56]. A common obstacle is the host’s preexisting immunity to certain vectors that could mitigate the efficacy of the vaccine [39]. The use of alternative vectors that have low seroprevalence in the host may circumvent anti-vector immunity [57,58,59]. The number of viral vector vaccines approved thus far for human use is still limited. The first viral-based vaccine approved for human use was Ervebo^®^ (Merck & Co., Kenilworth, NJ, USA), a vaccine against Ebola based on a recombinant vesicular stomatitis virus with a deletion in the envelope glycoprotein replaced with the Zaire Ebola virus (ZEBOV) surface glycoprotein (rVSV∆G-ZEBOV-GP). A second Ebola vaccine was also approved consisting of two components, Zabdeno^®^ that is given first and Mvabea^®^ that is administered approximately eight weeks later as a booster (Janssen; Johnson & Johnson; New Brunswick, NJ, USA). Zabdeno^®^ (Ad26.ZEBOV) is a recombinant Adenovirus type 26 (Ad26) encoding the ZEBOV glycoprotein while Mvabea^®^ (MVA-BN-Filo) is a recombinant Modified Vaccinia Ankara Bavarian Nordic Virus encoding the ZEBOV glycoprotein. Furthermore, four viral vector vaccines were recently approved in different countries for treatment of pandemic COVID-19 disease. Vaxzevria^®^ (AstraZeneca, Cambridge, UK and Oxford University, Oxford, UK) is based on a replication-deficient chimpanzee adenovirus (ChAdOx1) that contains the genetic material of the SARS-CoV-2 virus spike protein (ChAdOx1-SARS-CoV-2). COVID-19 Vaccine Janssen (Janssen, Johnson & Johnson) is based on a recombinant replication-incompetent adenovirus type 26 (Ad26) that contains the sequence that encodes the spike protein (S) of the SARS-CoV-2 (Ad26.COV2-S). Sputnik V (Gamaleya National Research Institute of Epidemiology and Microbiology) is a non-replicating heterologous recombinant adenovirus (rAd)-based vaccine that combines a recombinant adenovirus type 26 (rAd26) for the first dose and a recombinant adenovirus type 5 (rAd5) for the second dose, both carrying the sequence that encodes the spike protein (S) of the SARS-CoV-2. Ad5-nCoV (CanSino Biologics, Tianjin, China) is a non-replicating viral vector vaccine based on a recombinant adenovirus type 5 (Ad5) that contains the sequence that encodes the spike protein (S) of the SARS-CoV-2. On the contrary, viral vector vaccines represent the most common engineered veterinary vaccine type. Several poxviruses, including fowlpox and canarypox, have been used for the development of several licensed vaccines for veterinary diseases, such as Newcastle disease, avian influenza, equine influenza, rabies, FeLV and canine distemper [60,61]. A comprehensive analysis of the status of poxvirus vaccines under development for veterinary and human use is described by Sánchez-Sampedro and colleagues [62]. Another variant of the vectored vaccine approach is use of a live-attenuated viral pathogen as vector to induce protection against two diseases at once. 

### 3.3. Nucleic Acid–Based Vaccines

Nucleic acid vaccines are based on DNA cloned into a delivery plasmid or the direct injection of messenger RNA (mRNA) to express antigens into the host cells [38]. The endogenous protein synthesis mimics a natural infection eliciting both cellular and humoral responses [38]. The synthetic nature of this method also results in a high safety profile and a rapid manufacturing ability [63]. In addition, circumventing the need of cell cultures for protein expression results in lower manufacturing costs. Nevertheless, although mRNA vaccines are produced by in vitro synthesis through enzymatic processes, they rely on DNA as starting material. Plasmid DNA are typically amplified in *E. coli* and purification steps are also required. The recent production of COVID-19 vaccines has increased the demand of DNA production and current processed may become a bottleneck. The DNA industry is looking toward innovations in cell-free processes to meet the growing demand. Companies such as DNA Script (Paris, France) and Touchlight (Hampton, UK) are developing their own technologies to achieve this goal. In contrast to mRNA, DNA has an increased stability, avoiding the need of frozen storage and low temperature transportation. Nevertheless, this method generally requires further delivery formulations or specific delivery devices to reach good immunogenicity [64]. Promising results achieved in animal models with DNA vaccines were hardly reproduced in clinical studies, showing lower immunogenicity [65,66]. These results in bigger animals and humans are probably due to the difficulty to upscale the DNA vaccine amounts used in small animals [67], leading to lower amounts of expressed antigens. To get inside the cells, DNA molecules require a physical delivery method, commonly based on electroporation technology and DNA molecules that successfully enter the cell and also need to cross the barrier of the nuclear membrane to be transcribed [68], decreasing the amount of effective DNA molecules. Therefore, improvements in delivery methods are key to increase the amount of DNA that finally is transcribed in the nucleus to generate an appropriate amount of antigen to prompt an effective immune response. Although the potential of DNA vaccines to integrate into the host cell genome was one of the primary safety concerns [69], multiple studies have confirmed that the rate of genomic integration is negligible, demonstrating that they have an extremely low probability of genome integration [70]. Furthermore, the manufacturing process for DNA plasmid vaccines is well-established, and DNA vaccines have already been approved for veterinary purposes. Some examples are Apex-IHN^®^ (Novartis Aqua Health, Basel, Switzerland), which protects Atlantic salmon from infectious hematopoietic necrosis (IHN); West Nile–Innovator^®^ (Zoetis, Parsippany, NJ, USA), an equine vaccine that protects from West Nile virus (WNV); and ONCEPT^®^ (Boehringer Ingelheim, Ingelheim, Germany), a cancer vaccine that is used to treat canine malignant melanoma (CMM) [12]. 

Regarding human vaccines, mRNA vaccines have emerged as a promising vaccination strategy to face pandemic infectious diseases such as the recent COVID-19 disease. This platform led to a rapid vaccine development, and two novel mRNA vaccines were approved: Comirnaty^®^ (Pfizer, New York, USA and BioNTech, Mainz, Germany) and COVID-19 Vaccine Moderna (Moderna, Cambridge, MA, USA), both of them nucleoside-modified messenger RNA produced by using a cell-free in vitro transcription from the corresponding DNA templates, encoding the viral spike (S) protein of SARS-CoV-2.

RNA vaccines possess important advantages, including efficacy, safety and the potential for rapid, inexpensive and scalable production [71]. They lack the risk of insertional mutagenesis because they are based on the delivery of mRNA molecules directly into the cytoplasm of host cells, where antigens are transcribed, without the need to be directed to the cellular nucleus [40]. However, mRNA require a delivery system that not only protects the nucleic acid from degradation but also assists their entry into the cells. These molecules are large to diffuse into cells and their dense negative charge electrostatically repulses the anionic cell membrane, avoiding their uptake [72]. Recent COVID-19 mRNA vaccines are based on the use of lipid nanoparticles (LNPs) made of ionizable lipids as delivery vehicles [73]. Ionizable amine groups are positively charged at low pH, interacting with the anionic mRNA during particle formation and also facilitating membrane fusion during internalization [74,75]. A key aspect of LNPs that makes them different from liposomes is the presence of lipids in the core; however, data from several studies indicate that water is also present to some extent [76,77,78]. Moreover, mRNA-LNPs are endocited by cells and mRNA molecules are released into the cytoplasm. Lipid nanoparticles usually contain a helper lipid to promote cell binding, cholesterol to fill the gaps between the lipids and a polyethylene glycol (PEG) to reduce opsonization by serum proteins and reticuloendothelial clearance [72,79]; however, the final formulation needs to be optimized for each purpose.

Several strategies have been developed to solve the common issues of mRNA vaccines related to in vivo stability, translation capacity and reactogenicity of mRNA. Stability and translational efficiency can be improved by the optimization of the regulatory regions of mRNA: 5′ cap, poly-A tail and untranslated regions (UTRs) [73]. Furthermore, maximization of GC (guanine–cytosine) content in combination with codon optimization by selection of frequent codons in the coding region also allows to enhanced stability and translation [80,81]. The secondary structure of mRNA is also of great importance and it can be stabilized by changing the primary sequence through codon optimizations and bioinformatics prediction. Alternatively, it has been reported that the incorporation of naturally occurring modified uridines, such as the use of 1-methyl-pseudouridine (m1Ψ) instead of uridine, induces global changes in the secondary structure of mRNA which correlates with high protein expression [82]. Furthermore, modification of uridines is also an efficient way to minimize reactogenicity of mRNA, decreasing the cellular recognition by RNA-binding proteins involved in the innate immune response to foreign mRNA [73].

### 3.4. Protein-Based Vaccines

Protein vaccines use peptides or proteins as antigens to induce an immune response. As previously described, protein antigens can be obtained from the pathogen that causes the infectious disease as a natural source, either in their whole form or as derived split-products. Although protein vaccines produced from natural infectious agents still fulfill an important role, they show some disadvantages, as production and purification processes are costly, time-consuming and require the growth of large quantity of pathogens [38]. In addition, some viruses cannot be cultivated efficiently or are risky to cultivate in large scale, making difficult the production from natural sources. In other cases, human proteins, such as certain protein factors, could also be required for treatment of non-infectious diseases, such as hypertension and cancer [83]. As an alternative to natural sources, protein subunit vaccines can also be produced as heterologous proteins in recombinant systems. Production through well-established expression systems, including recombinant bacteria, yeast, insect cells or mammalian cells, is considered to be more cost-effective [83] and safe compared with natural sources. Nevertheless, the process to find the appropriate antigenic component to produce an effective immune response is time-consuming [8,84]. Although full-length proteins can induce the development of antibodies against multiple epitopes and they tend to maintain their native conformation [39], they also increase the possibilities of inducing nonspecific cross-reactive antibodies [85]. Since only specific amino acid sequences of the full-length antigen are responsible for effective immune responses, selected immunogenic peptides mimicking B- and T-cell epitopes have also been proposed as vaccine candidates [85]. Consequently, optimizing amino acid sequence is of great importance to promote epitopes associated with neutralizing antibodies, that block pathogens and confer protection, while reducing epitopes associated with non-neutralizing antibodies [86]. Subunit vaccines tend to generate low immunogenicity in comparison with traditional vaccines due to relative small sizes and low valences of the antigens or epitopes [83]. Moreover, whole-organism vaccines, whether inactivated, attenuated or from a closely related species, do not expose just one copy of an antigen, because they contain multiple copies of each antigen, as well as other immunostimulatory molecules, and with subunit protein vaccines, this effect does not occur [8]. Many approaches have been explored to develop effective subunit vaccines, including the use of adjuvants to stimulate the immune system and the use of nanotechnology to generate effective antigen display systems [87]. The addition of adjuvants can help the vaccine to be more immunogenic, induce a stronger humoral or cellular immune response, increase antigen processing by APC, decrease the total amount of vaccine that needs to be injected or aid in the development of a long term memory response [88]. Marketed protein-based vaccines for human and veterinary use, classified by production system, are listed in Table 1 and Table 2, respectively.

## 4. Nanotechnology Applied to Subunit Vaccines

An emergent area in the development of vaccines is the use of nanotechnology that works with a wide range of materials to generate effective antigen display systems. These non-replicative and organized structures on a nanoscale of 1–100 nm can be produced by recombinant expression [129] or chemical synthesis [130]. While biological nanoparticles have thus far predominated nanovaccine trials, inorganic and chemically synthesized nanoparticles, including metals [131] and synthetic polymers [132], are also being explored in preclinical studies. Some of the biological approaches to generate effective antigen display systems are the use of VLPs and subviral particles, ferritin cages, vault particles, encapsulins, liposomes, virosomes and outer membrane vesicles, among others [8].

### 4.1. VLPs and Small Subviral Particles

Virus-like particles (VLPs) mimic the natural process of viral capsid self-assembling through recombinant technology to generate particles that exhibit similar structural and antigenic properties of their authentic viruses [83]. Small subviral particles can also be constructed by generating truncated viral structural proteins [12]. They resemble the size and shape of viruses but do not contain any viral genetic material, meaning that they are not infectious [50]. VLPs offer the advantage of being repetitive antigen display systems and it has been demonstrated that antibody titers increase when antigens are displayed repetitively [50,133] due to an increase of crosslinking B-cell receptors leading to B-cell activation [15,134,135]. In addition, VLPs possess self-adjuvanting properties due to their particulate structure and optimal size for uptake by antigen presenting cells [136,137]. Thus, they are efficiently processed by APCs, leading to activation of T cells, making them an interesting tool for increasing the immunogenicity of antigens [50]. VLPs can be antigens themselves, meaning the protein that assembles into the VLP is the antigen of interest. This is the case for VLP vaccines such as HBV, HPV, PCV2 or PPV. Alternatively, VLP can be used as a scaffold or carrier for the delivery of heterologous antigens as a platform technology. Well-reported platforms based on self-assembling proteins acting like carriers include HPV L1 [138] and hepatitis B core [139] or surface antigens [140,141], among others. VLPs are usually produced in bacteria, yeast or insect cells as expression system [142]. Expression of large genetically fused antigens is challenging, and strategies such as linker designs [143], antigen titration [106,143,144], split-intein conjugation [145,146,147] and tandem core fusion strategy [148] are implemented to enable ease of large antigen modularization. The vaccine RTS,S/AS01 Mosquirix^®^ (GSK, Londres, UK) against malaria [104,106] is one of such combination vaccines that has reached the market. It consists of hepatitis B surface antigen (S) VLPs containing a portion of *Plasmodium falciparum*–derived circumsporozoite protein (RST). Further VLP vaccines have been approved for human use, such as Recombivax HB^®^ (Merck & Co., Kenilworth, NJ, USA) and Engerix^®^-B (GSK, Londres, UK) against hepatitis B virus (HBV), Gardasil^®^ (Merck & Co., Kenilworth, NJ, USA) and Cervarix^®^ (GSK, Londres, UK) against human papillomavirus (HPV) and Hecolin^®^ (Innovax, Xiamen, China) against hepatitis E virus (HEV).

### 4.2. Ferritin Cages

Ferritin cages are protein assemblies derived from ferritin, an iron metabolism protein present in bacteria, animals and plants that, under normal conditions, self-assemble into a spherical particle of 24 monomers with octahedral symmetry containing an open central cavity [149]. The overall diameter of the particles is 12 nm and the cores have a diameter of 8 nm [150]. Although ferritin cages seem excellent candidates for repetitive display antigens, the main limitation is their rigid assembly. Thus, if an antigen needs to be presented in a certain conformation to be immunogenic, ferritin cages may not be able to present it in the most immunogenic form [8].

### 4.3. Vault Particles

Vaults are naturally occurring nanoparticles found widely in eukaryotes as a 70 nm organelle-like structure that is composed of the major vault protein (MVP), telomerase associated protein-1, poly ADP-ribose polymerase (PARP) and non-coding RNAs [151]. They are not immunogenic and do not lead to the development of autoimmune responses making them a good vaccine delivery system [152]. Nevertheless, their biggest weakness is that antigens, instead of being exposed at the surface of particles, are contained within the vault cavity, lacking the direct stimulation of B cells responsible for humoral responses [8].

### 4.4. Encapsulins

Recently, a new class of prokaryotic compartments, known as encapsulins or protein nanocompartments, has attracted the attention in the field of nanotechnology as systems for delivery being of great interest as potential antigen display systems. Encapsulin protein self-assemblies to form an icosahedron made of 60 identical subunits, with a diameter of 25–42 nm, differing in size depending on the bacterial source [153]. Foreign antigens can be either encapsulated in the nanocompartment as cargo or exposed at the surface of the nanoparticles. A recent work demonstrated that when Epstein–Barr Virus glycoprotein 350/220 (gp350) was expressed at the surface of encapsulins, potent neutralizing antibodies were elicited in mice and non-human primates, increasing neutralization 10- to 100-fold compared to soluble gp350 [154].

### 4.5. In Silico Designed Nanoparticles

An alternative to naturally occurring nanoscale assemblies are in silico rationally designed particles, able to self-assemble into nanoscale assemblies to improve antigen display. Examples of successfully designed two-component nanoparticles as vaccine candidates include BG505 SOSIP-I53-50 nanoparticles for HIV [155], DS-Cav1-I53-50A nanoparticles for Respiratory Syncytial Virus (RSV) [156] and BG505 SOSIP–T33_dn2 nanoparticles for influenza, HIV and RSV [157].

## 5. Traditional Platforms for Protein Vaccine Manufacturing

Bacteria (*E. coli*), yeast, insect cells and mammalian cells have been used traditionally as platforms for production of recombinant proteins. Regarding vaccine manufacturing, according to the number of approved recombinant protein vaccines for human use (Table 1), yeast and insect cells are the most extensively used production platforms for this industry. Furthermore, they are also the preferred platforms for VLP manufacturing purposes. In the case of animal health, insect cells are the dominant platform for recombinant vaccine manufacturing (Table 2).

### 5.1. Bacteria (E. coli)

The first recombinant expression system was established using *E. coli* bacteria, the microbe used to develop the early concepts of molecular biology [38]. This expression system is characterized by high protein yield, fast growth rate, high productivity, short production times and low manufacturing cost. The price of culture medium and upstream processing for this platform is significantly lower when compared to eukaryotic systems such as insect or mammalian cell cultures. However, this system also has some disadvantages such as the lack of eukaryotic post-translational modifications that can lead to the expression of misfolded, insoluble or nonfunctional proteins. Complex antigens requiring post-translational modifications (e.g., glycosylation and multimer assembly) cannot be correctly produced in this platform [27]. Engineered bacteria able to induce specific post-translational modifications can solve this problem, but it makes the process more difficult [158]. Furthermore, the removal of contaminant endotoxins generated in this system, mainly lipopolysaccharide, should also be taken into account in the manufacturing process. Despite active investigation of novel systems, *E. coli* remains the dominant bacterial strain in use [159] because is easy to engineer and adapt to new constraints, such as antibiotic-free selection [160,161]. Furthermore, development of new bioinformatic tools has enabled prediction of potential expression issues, as could be protein solubility upon overexpression [162]. Regarding human health, the first vaccine produced in *E. coli* was approved in 2012 for market commercialization in China. Hecolin^®^ (Innovax, Xiamen, China) is the first world vaccine against Hepatitis E virus and is based on the ORF2 capsid protein of HEV that is refolded after expression and self-assembly into VLP [89,90]. Recently, the FDA has approved Hecolin^®^ to enter a Phase 1 clinical trial in the United States (NCT03827395). A recombinant HPV type 16/18 bivalent vaccine produced in *E. coli* (Cecolin^®^; Innovax, Xiamen, China) has also been approved recently for marketing in China. In addition, a recent study has shown that *Plasmodium vivax* antigens expressed in *E. coli* induced humoral and cellular immune responses in mice, showing promising results as potentially useful vaccine candidates against *P. vivax* malaria [163]. One of the first recombinant veterinary vaccines to be successfully produced in *E. coli* was Leucogen^®^ (Virbac, Carros, France), a purified recombinant p45 FeLV-envelope antigen that was derived from the gp70 surface glycoprotein of the FeLV [116,117]. Another example is Letifend^®^ (LETI Pharma, Barcelona, Spain), a recombinant vaccine against Canine leishmaniasis based on a recombinant protein Q from *Leishmania infantum* also expressed in *E. coli* [118].

### 5.2. Yeast

Yeasts are attractive hosts for recombinant protein expression due to their fast growth, high protein yield, simple handling, low production costs and industrial production knowledge, advantages that have in common with prokaryote systems. Moreover, they also possess some advantages of eukaryotes systems such as the capacity to perform post-translational modification of proteins in a manner similar to that used by higher eukaryotic cells, allowing recombinant proteins to be more likely correctly folded [38]. All these advantages make yeast a cost-effective production system when compared to higher eukaryotic systems such as animal cells. Regarding safety, they also avoid the endotoxin problem that is associated with bacterial expression systems and viral contamination of mammalian expression systems [164]. Yeasts also face some challenges, as their glycosylation pattern is distinct from that carried out by mammalian cells, leading to hyperglycosylation (excess of mannose residues) of recombinant proteins. To change the high-mannose profile of yeast to the human-type complex glycan pattern, the glycosylation pathways have been genetically altered in some strains, such as *S. cerevisiae* [165,166], *H. polymorpha* and *Pichia pastoris* (*P. pastoris*) [167,168]. Moreover, yeasts are also being engineered to overexpress chaperones with the aim to enhance secretion and folding of recombinant proteins [169,170,171]. *S. cerevisiae* is the most extensively used yeast host for heterologous protein production and several vaccines have already been successfully produced in this system. Recombivax HB^®^ (Merck & Co.) was the first recombinant vaccine approved for human use and is directed for treatment of hepatitis B, based on HBV surface antigen (HBsAg) that self-assembles into VLPs [91,92]. HBsAg was previously produced in *E. coli* but it was not immunogenic, which led to a search for a different expression host [172,173,174]. The second example is a vaccine directed against HPV and is based on the structural L1 protein that also self-assembles into VLPs (Gardasil^®^, Merck & Co.) [99,100,101]. Although many heterologous proteins have been successfully expressed in *S. cerevisiae*, this system also faces some issues related with low yields of protein expression and hyperglycosylation, which may result in differences in immunogenicity, diminished activity or decreased serum retention of the foreign protein [175]. Non-conventional strains such as *P. pastoris* are being explored as alternative hosts to surmount the abovementioned limitations.

### 5.3. Insect Cells

Insect cells have been widely used for recombinant protein expression because of their capacity to produce high levels of proteins and to perform post-translational modifications, including glycosylation, phosphorylation, disulfide bond formation and protein processing required for the biological activity of many complex proteins [176]. Although stable transformed insect cell lines can be generated, the most extended system is the transient expression driven by recombinant baculovirus infection. Baculovirus expression vector system (BEVS) is one of the most well-known and used systems for large-scale production of complex proteins. Baculoviruses are insect pathogens and do not represent a human health risk. They can be easily modified to incorporate multiple additional foreign genes, and *Autographa californica* multiple nucleopolyhedrovirus (AcMNPV) is one of the dominant vectors. The protein of interest is usually produced under the control of the polyhedrin (*polh*) promoter, one of the strongest promoters known in nature [177], which can express foreign proteins up to 50% of total cell proteins [38]. The *p10* promoter that is active at an earlier time post-infection is also widely used; however, in general, it reaches lower levels than the *polh* promoter [178]. The use of multiple promoters makes possible the expression of several proteins simultaneously in a single infection. The most commonly used lepidopteran insect cell lines are Sf21 and Sf9 derived from *Spodoptera frugiperda* and the BTI-TN-5B1-4 cell line (High Five™) derived from *Trichoplusia ni* [159]. These cells grow in suspension without requirement of CO_2_ leading to a feasible scale up of protein production. Insect cells are less demanding and grow to higher densities than mammalian cell lines, but they also demand expensive culture medium and the use of bioreactors, which increases the production costs. Expression levels as high as 1 g per liter could be obtained; however, levels can vary considerably from 1 to 600 mg depending on the antigen [38]. On the other hand, insect cells have also some limitations as they cannot synthesize mammalian-specific complex glycan structures. N-glycans from insect cells are not processed to terminally sialylated complex-type structures but are instead modified to paucimannose structure [179]. This issue has been addressed by genetic modification of either insect cells [180,181,182,183] or baculoviruses [184] to include genes encoding N-glycosylation functions. In addition, although the growth rate of insect cells is higher than mammalian cells, it is still lower when compared to yeast or bacteria, and production requires more expensive culture media and longer times than those required for microbial systems.

Recently, vaccines produced in insect cells have been commercialized, demonstrating that this form of production has potential as a commercial manufacturing technology [185]. The first commercially available veterinary vaccines produced in insect cells were classical swine fever virus (CSFV) vaccines (Bayovac^®^ CSF E2; Bayer, Leverkusen, Germany and Porcilis Pesti^®^, Merck & Co., Kenilworth, NJ, USA), based on the E2 antigen [119,120,121]. In addition, veterinary vaccines against PCV2 based on the ORF2 protein were also approved: IngelVac CircoFLEX^®^ (Boehringer Ingelheim, Ingelheim, Germany) [122,123,124], Porcilis^®^ PCV (Merck & Co.) [126], Circumvent^®^ PCV2 (Merck & Co.) [125] and CircoGard^®^ (Pharmgate, Wilmington, NC, USA) [127]. A vaccine against porcine parvovirus, ReproCyc^®^ ParvoFLEX (Boehringer Ingelheim, Ingelheim, Germany), based on the viral protein 2 (VP2) was also approved for commercial use in 2019 by the EMA [128]. The first human vaccine produced in insect cells, Cervarix^®^ (GSK), was licensed by the European Medicines Agency (EMA) in 2007 and by US Food and Drug Administration (FDA) in 2009 [108,109]. Cervarix^®^ is a bivalent human papilloma virus vaccine (HPV 16 and 18) based on the expression in High Five™ cells of the major capsid protein L1 of HPV that self-assembles to form VLPs [107]. The second product for human use licensed by the FDA was Provenge (Dendreon, Seal Beach, CA, USA) [186,187], an autologous prostate-cancer therapy. The antigen PA2024, a fusion protein of prostatic acid phosphatase (PAP) and granulocyte–macrophage colony-stimulating factor (GM–CSF) produced in Sf21 cells is used to stimulate dendritic cells from each patient ex vivo that will be further used as a cellular vaccine. Moreover, recombinant influenza vaccines based on the hemagglutinin (HA) surface antigen produced in insect cells developed by Protein Sciences (now owned by Sanofi Pasteur, Lyon, France) have also been approved: Flublok^®^ [110,111] and Flublok Quadrivalent^®^ (Supemtek^®^ in EU market) [112].

Alternative insect cell line as *Drosophila* Schneider line 2 (S2 cells) is also being used as a stable, nonviral and nonlytic expression system able to successfully produce difficult to express proteins [159]. They are also suitable for cultivation, using batch, fed-batch and continuous cultivation techniques [159]. This system is being explored for the development of vaccine candidates against dengue virus [188] by Merck & Co., West Nile virus [188] by Hawaii Biotech (Honolulu, HI, USA) and also two malaria vaccines from the Jenner Institute (University of Oxford) and Copenhagen University, respectively [188].

### 5.4. Mammalian Cells

Mammalian cells are extensively used to manufacture diverse immuno- and biotherapeutic molecules, due to their high and robust productivity and secretion of complex molecules in serum-free medium and their ability to perform complex post-transcriptional modifications [189], such as precise glycosylation. Some of the limitations of this system are that the speed of production is low, the cost is high (culture media and fermentation system) and there is also a potential risk for endogenous virus contamination [159]. To avoid virus contamination, biopharmaceutical manufacturing processes follow measures based on prevention, in-process control and clearance according to regulatory guidance [190]. Prevention of virus entry is achieved by selecting low-risk starting and raw materials and performing manufacturing controls. In-process testing of materials validate the absence of viruses, assuring lot rejection in case of contamination. Finally, a step of virus clearance for virus inactivation and/or removal is performed to assure the absence of viral contamination in the final product.

More than 60% of the currently available immunotherapeutic molecules produced in mammalian cells are monoclonal antibodies [159]. Regarding vaccine manufacturing, mammalian continuous cell lines (CCLs) are used for virus propagation in the production of virus-based vaccines. Vero (African monkey kidney epithelial) cell line is one of the most widely used for this purpose and has been used for production of polio and rabies virus vaccines [191]. The use of mammalian cells for the production of recombinant protein vaccines is also possible, but costs of manufacture are high and expression levels can be lower than those achieved by using alternative expression systems. Nevertheless, this system could be useful for the expression of complex vaccine candidates. 

Two of the most commonly used mammalian cell lines for recombinant protein expression are Chinese Hamster Ovary (CHO) and Human Embryonic Kidney 293 (HEK293) cells. The CHO cell line was derived from a biopsy of the ovary of the Chinese hamster while the HEK293 cell line was generated by transfection of a human primary embryonic kidney cell culture with sheared DNA of adenovirus type 5 (Ad5). Both cell lines can be grown in suspension or as adherent cultures; however, suspension cultures are the preferred choice at the industrial scale, as they are easier to scale up and to adapt to automated processes. Expression of foreign genes can be achieved either by transient or stable expression. For large amounts of protein production, stable cells are preferred, as they provide high yields with consistent quality. Taking into account that the generation of stable cell lines is time-consuming, transient expression that results in high levels in short periods is usually the method of choice for rapid protein production during Proof-of-Concept stages. HEK-293 cells are often used for transient expression due to its high transfection efficiency while CHO cells are more commonly used for stable cell line expression. HEK-293 cells show different glycosylation patterns from those proteins made in CHO cells, a feature to be considered during development if the host cell type required to be modified, as it could affect the biologic activity of the proteins expressed.

Development of recombinant cell lines for stable protein expression relies on the integration of a plasmid containing the sequence of interest into the genome. When cells are transfected by chemical reagents or by electroporation, non-homologous recombination occurs in a small portion of the cell; however, the efficiency of this process is very low. To achieve a stable expression, it is necessary to integrate the genes of interest at sites of the genome that are transcriptionally active, that do not experience gene silencing [192] and that are not susceptible to genetic rearrangement [193]. Viral vectors can be used as tools for this purpose, as they target transcriptionally active regions of the genome to integrate their DNA. Lentiviral vectors can transduce CHO cells with high vector copy and remain stable for many months [194,195]. Alternative systems are non-viral adeno-associated virus (AAV) system, based on the AAV machinery [193] or the novel viral vector system Semliki Forest virus (SFV), developed from alphaviruses [196].

As the genome sequence of several production cell lines is already available, editing of genomic sequences with nucleases is also possible. The CRISPR/Cas system has been successfully used for genome editing in CHO during cell-line development [197], and site-specific integration of a therapeutic gene has also been reported [198].

CHO cells are widely used for production of recombinant glycoprotein therapeutics due to their high productivity (1–10 g per liter), genetic stability and ability to grow in large-scale suspension culture [199,200,201] in serum-free medium. However, many recombinant proteins, including monoclonal antibodies, antibody fusion proteins and IFN-γ, are partially degraded or proteolyzed by endogenous CHO cell proteases during the cell culture or recovery process [202,203,204,205,206,207]. Novel approaches to solve proteolysis and enhance glycosylation for production of HIV envelope proteins as vaccine candidates are being explored [208]. The HEK293 cell line is also a platform of interest for production of recombinant viral vectors and vaccines at the industrial scale, because the cells are easy to grow in suspension and can be adapted to serum-free medium. Current developments of human vaccines using HEK293 cells include the expression of glycoproteins for the treatment of infectious diseases, such as rabies [209] or Ebola virus [210]. A recent study of a vaccine candidate against *Trypanosoma vivax* showed that an invariant antigen expressed in HEK293 cells was able to induce protective immunity in mice [211], showing promising results as a vaccine candidate. A Zika virus vaccine candidate expressed in HEK293T cells (a HEK293 cell line derivative transfected with a SV40 expression construct) was reported to protect against ZIKV challenge in pregnant mice [212].

Although the application of mammalian cells to vaccines development is still limited, already there is a human vaccine produced with CHO cells that is approved for human use. Shingrix (GSK, Londres, UK) is a herpes zoster vaccine based on varicella zoster virus glycoprotein E (gE) [113,114,115].

## 6. Alternative Platforms for Protein Vaccine Manufacturing

Traditional vaccine platforms meet market demands for some application but costs of manufacturing, scale capability and times required for development make difficult to accomplish other market requirements. Conventional vaccine-manufacturing technologies are limited to producing only one vaccine or a very narrow range of vaccines; thus, individual manufacturing processes need to be developed for each vaccine, making vaccine-manufacturing-process development costly and time-consuming [213]. It is estimated that developing a vaccine from concept to market costs $200 million–$500 million and takes 5–18 years [214,215,216,217,218]. Additionally, it costs an estimated $50 million–$700 million to construct, equip and commission a vaccine manufacturing facility, taking on average 7 years [215,216,219,220], while the manufacture process of a vaccine ranges between 0.5 and 3 years [215,221]. Platform technologies allow the standardization of upstream and downstream processes, given that the platform base remains unchanged. Certain processes would require optimization but this system provide flexibility and possibility for multi-product facilities [44]. Flexible, rapid and low-cost vaccine development and manufacturing technologies are required to meet vaccination demands in low- and middle-income countries and to face possible future epidemics. Tight profit margins associated with intensive animal production also reveal the necessity to produce inexpensive vaccines for animals [12]. The lower cost for veterinary vaccination in the range of cents per dose [222] compared with a typical cost range of $20–$130 per dose for recombinant human vaccines (http://www.cdc.gov/vaccines/programs/vfc/cdc-vac-price-list.htm, accessed on 1 June 2021), evidences that low-cost vaccine development and manufacturing technologies are critical for animal health. Therefore, there is a pressing need of alternative production platforms that can overcome all the existing limitation in vaccine production. Within the development of alternative systems, two main strategies can be considered: the rational engineering of existing systems and the development of completely alternative hosts. The engineering approach may be useful to improve the quality of the expressed protein such as the glycosylation profile, but in terms of productivity the improvements will meet limits due to the physiological capacity of the systems. In this case, the use of new hosts with a naturally extended capacity for very high protein production and secretion could offer higher potential [159]. 

### 6.1. Non–E. coli Bacterial Systems

Some alternative Gram-negative hosts have been explored as expression systems, but their utility still remains marginal in comparison with *E. coli*. One of them is *Pseudomonas fluorescens* [223], for which a complete toolbox for protein expression and strain selection is available. Another host that is considered outstanding for its high metabolite output is *Ralstonia eutropha* [224]; however, tools for protein expression in this system are still limited. Regarding Gram-positive hosts, many Lactic Acid Bacteria (LAB), such as *Lactobacillus* and *Bifidobacterium*, have been extensively used in the food industry as probiotics. They represent an attractive tool for oral vaccine production and delivery because of their GRAS status, reported adjuvant properties, mucoadhesive ability, easy genetic manipulation and the availability of well-defined industrial production processes [225,226]. Effective mucosal immunogenicity and protection after oral and nasal vaccination have been reported using *Lactobacillus* strains expressing antigens against several viral and bacterial pathogens [227,228,229,230,231,232]. Nevertheless, vaccine strains cannot be considered avirulent, even if they could be listed as GRAS, due to potential transfer of antibiotic selection markers among microbes [233,234]. To solve this issue, a carrier system called Gram-positive enhancer matrix (GEM) based on non-recombinant *Lactococcus lactis* bacteria was recently developed. It is composed of the rigid peptidoglycan (PGN) cell wall resulting in a non-living particle that preserves the shape and the size as the original bacterium [235]. GEMs can be used both mixed with vaccine antigens as adjuvants or as antigen protein carriers with the antigens bounded to their surface [34].

### 6.2. New Approaches in Yeast Platform

In addition to *S. cerevisiae* other non-conventional strains are also of great interest for production of therapeutic proteins such as *Pichia pastoris* (*P. pastoris*), *Saccharomyces boulardii* (*S. boulardii*) and *Kluyveromyces lactis* (*K. lactis*) [236]. A comparative of advantages and disadvantages of different yeast species as hosts for expression of heterologous proteins has been extensively discussed by Vieira Gomes and colleagues [237]. *P. pastoris* is one of the most studied non-conventional strains for the production of recombinant protein antigens for human vaccines [238,239] because is capable of performing human-like post-translational modifications, showing weaker hyper-mannosylation than those performed by *S. cerevisiae* [164]. Expression in *P. pastoris* also offers advantages for secreted recombinant proteins as it grows on a simple mineral media and secretes only low levels of endogenous proteins. Therefore, the heterologous protein can comprise the major portion of the total protein in the medium, leading to an easier purification process [240]. Antigens from virus, protozoa, bacteria, nematode and tick expressed in different species of yeast, including *P. pastoris*, have been extensively reviewed by Kumar and colleagues [241]. Recently, it has been reported that Zika virus envelope protein (80E) expressed in *P. pastoris* is able to elicit potent ZIKV-neutralizing antibodies in mice conferring significant protection in vivo [242]. SARS-CoV-2 recombinant receptor-binding domain candidate vaccine expressed in *P. pastoris* has also been reported to be able to stimulate virus neutralizing antibodies and T-cell immunity in mice [242].

New approaches in recombinant yeast vaccine technology include the use of inactivated whole yeast cells [243] or antigen display at the yeast cell surface [242] as vaccination strategy. These technologies are of great interest for the development of oral vaccines at low-cost because no purification is needed. In addition, they could serve as a universal technology platform for vaccine development. The GRAS status of some strains, such as *S. cerevisiae*, and the properties of the cellular wall to protect the antigens across the gastrointestinal tract make engineered yeast an attractive vaccine delivery system [27,244]. Moreover, this delivery system could also enhance immunogenicity of vaccines due to the adjuvant activity of β-glucans on the yeast cell wall that have demonstrated immunomodulatory and adjuvant effects through the binding of innate pathogen receptors on macrophages, DC and neutrophils [245]. Some preclinical studies based on orally administrated *S. cerevisiae* displaying antigens on its surface for different infectious agents, such as influenza, showed that this delivery system is able to induce both humoral and cellular responses in mice [242]. Current developments include the expression of the spike protein of SARS-CoV-2 on the surface of *S. cerevisiae* as vaccine candidate, showing promising results in mice [246].

### 6.3. Transgenic Animals

Complex proteins can be produced by generating transgenic animals not only in mammalian species such as goat, sheep, rabbit or pig capable of expressing proteins in milk, but also in transgenic hens [247,248] or even ostriches [249] that can produce proteins in egg albumen. The selection criteria for the transgenic animal is of primary importance and includes parameters to be considered such as annual dairy production, reproductive performance or age of sexual maturity [250]. Safety issues, such as cross-contamination should also be considered, and animals should be able to be maintained in pathogen-free conditions to prevent contamination [251]. Moreover, the ongoing ethical and societal debate around the use of transgenic animals includes concerns related to animal welfare and the use of genetically modified organisms (GMOs) [252,253]. Furthermore, the investment required to develop an innovative platform that still poses a high degree of uncertainty, so, at present, the big companies prefer to improve the use of mammalian cells rather than explore transgenic animals [254].

Despite the relative complexity and lengthy timelines associated with the generation of transgenic animal bioreactors, there are some niche therapeutic areas where this system could be useful, including high-added-value markets requiring complex molecules for which other systems have failed or cannot fulfill demand [159]. The first recombinant protein approved for human use expressed in transgenic animals was a human antithrombin III (ATryn^®^; rEVO Biologics, Framingham, MA, USA) produced in goat milk. It was approved by the EMA in 2006 and by the FDA in 2009 for treatment of patients with hereditary AT deficiency [255]. This case reveals the potential of transgenic animals since a single genetically modified goat can produce as much antithrombin in a year as 90,000 blood donations [256]. In 2011 the EMA approved an additional biologic (Ruconest^®^; Pharming, Leiden, the Netherlands), a recombinant C1-esterase inhibitor for the treatment of hereditary angioedema produced in the milk of transgenic rabbits. Eggs from transgenic chickens are also a promising system for recombinant protein production. In 2015, the FDA and EMA approved Sebelipase alfa (Kanuma^®^; Alexion, Boston, MA, USA), an orphan drug based on a recombinant form of the enzyme lysosomal acid lipase (LAL) for treatment of lysosomal acid lipase deficiency (LAL-D) produced in egg whites. Although regulatory approval for novel platforms is challenging, the growing number of approved products from transgenic animals makes a significant contribution in terms of regulatory issues, demonstrating that these platforms can meet regulatory requirements.

While there are numerous examples of therapeutic molecules produced in transgenic animals, antigen production is still limited. Nevertheless, this system allows for the production of complex molecules with sophisticated post-translational modification and could be of great utility for expression of problematic antigens such as the malaria major surface protein (MSP-1), which has been already successfully expressed in transgenic goats for the production of a candidate vaccine [257].

### 6.4. Insects

Baculovirus expression vector system (BEVS) is not only limited to insect cells cultures; insect larvae or pupae can also be used for recombinant protein production. Industrial manufacturing using insect cells, as with any other cultured cells, requires artificial media and the use of expensive bioreactors. Protein expression using insect larvae as biofactories offers a cost-effective alternative to insect cells bioreactors and the capacity to produce large and multiple proteins with an easy scale-up production. This system offers several advantages, such as high protein expression levels with post-translational modifications of complex proteins. Although the glycosylation pattern differs from that of mammalian cells, as previously mentioned for insect cells, bioactive molecules can be efficiently expressed in this system. Nevertheless, downstream processing to obtain antigens from insects could be more complex than those established for culture cells. *Autographa californica* multiple nucleopolyhedrovirus (AcMNPV) and *Bombyx mori* nucleopolyhedrovirus (BmNPV) are the most extensively used vectors in the BEVS to produce heterologous proteins in insect larvae. The dominant species of insect larvae are the silkworm *Bombyx mori* (*B. mori*) and the cabbage looper *Trichoplusia ni* (*T. ni*). Production of human alpha-interferon (IFN-alpha) in larvae of the silkworm *B. mori* was published in 1985 [258] and since then many recombinant proteins have been properly expressed in insects. Recombinant feline IFN omega produced in silkworms (Virbagen^®^ Omega; Virbac, Carros, France) has been approved for feline calicivirus infections in Europe and Canada, while Interdog™, a drug composed of canine IFN gamma for the treatment of canine atopic dermatitis, was marketed in Japan in December 2005 [159]. Recently, an efficient application of a baculovirus–silkworm larvae expression system for PCV2 VLP production has been described [259]. *T. ni* larvae has also be reported as host for production of vaccine candidates against PCV2 [260], West Nile virus (WNV) [261], RHDV [262] and Influenza [263], among others. A comparative analysis of the expression of L1 HPV protein in *T. ni* showed that the yield obtained in larvae was 2.5 times the L1 production yield obtained in insect cells and 5 times the production yield obtained in transplastomic tobacco plants, referred as g of fresh biomass [264]. Yield of expression of up to 2 mg of recombinant protein per larvae has been reported for *T. ni* as expression system [262]. Protein expression in *T. ni* pupae has also emerged as an innovative platform and expression of several proteins have been reported, reaching productivities on the range of milligrams per infected pupa [265]. As with transgenic animals, a societal debate also comes up regarding the use of biotechnology in insects; however, this platform allows for heterologous protein expression with no need to genetically modify the host genome. Regarding good manufacturing practice (GMP) compliance, upstream processes related to insect growing, as in the case of whole-plant systems, require special attention to meet classical GMP criteria based on bioreactors.

Insect larvae and pupae have also attracted the interest in the field of oral vaccines and silkworm *B. mori* has been studied as a feasible delivery system for vaccines [266,267]. While the data collected so far support the possible use of baculovirus–silkworm vaccines as a promising edible vaccine platform, nowadays it is only approved for food ingestion in a few Asian countries [34].

### 6.5. Plant-Based Systems

Plants show high potential as bioreactors for recombinant protein production because they allow the production of properly folded complex proteins and represent an alternative, cost-effective eukaryotic system [159]. Plant expression platform diversity includes whole plants, suspension cells, hairy roots, moss, duckweed and microalgae [268]. Differential glycosylation respect to the native antigen should be taken into consideration when using this system [269]. Plant proteins lack the terminal galactose and sialic acid residues commonly found in animals and possess α-(1,3) fucose and β-(1,2) xylose that animal proteins lack. As a result, glycoproteins from plants can lead to immune reactions and alter pharmacokinetic properties [256]. Nevertheless, glycoengineering approaches to avoid plant glycosylation or add human glycosylation can be used when this factor is crucial for antigen functionality or safety [270]. Plants can be genetically engineered to transiently or stably express antigens in the nucleus or in the chloroplast. Nuclear expression offers high biosynthetic capacity and complex post-translational modifications can be performed, while chloroplast transformation offers high levels of transgene expression (up to 70% of total soluble proteins) [271,272] but possesses rudimentary prokaryote-like expression pathways, lacking post-translational modifications such as glycosylation [159]. Stable expression is preferred in order to obtain a stable genetic resource; however, this method is time-consuming [273] and can lead to low expression yields [274]. On the other hand, transient expression technology that uses either *Agrobacterium* or viral vectors is robust, quick and easy to manipulate [275] but is typically more unstable [276]. Furthermore, the production timeline between antigen cloning and large-scale production is very short, and it only uses the plant as a substrate, without the need to classify it as a Genetically Modified Organism (GMO). Some of the limitations of the use of plant expression systems for the production of vaccines are the long timelines involved with the establishment of transgenic plants, low expression levels and relatively weak efficacy [277], representing a challenge for economic feasibility. Recent advances in transient expression achieved by recombinant viral vectors, such as *Agrobacterium*-mediated expression systems including Icon Genetics’ magnICON expression technology (magnifection), has led to a great increase in yields of protein expression. By using this technology in *Nicotiana benthamiana*, high levels of proteins can be achieved in weeks. An example of vaccine generation is the production of personalized idiotypic vaccines for follicular lymphoma. Purified antibodies were chemically linked to the carrier protein keyhole limpet hemocyanin (KLH) to form a conjugate vaccine that was evaluated in a phase I safety and immunogenicity clinical study. Results showed that 82% of patients displayed a vaccine-induced, idiotype-specific cellular and/or humoral immune response. Another example is Zmapp monoclonal antibodies against Ebola, using the large-scale Rapid Antibody Manufacturing Platform (RAMP) and magnICON vectors. In preclinical studies, they showed to be able to rescue 100% of rhesus macaques when treatment was initiated up to 5 days post-challenge, improving the efficacy of any other therapeutics described so far [278]. Advantages of plants over microbial systems include manufacturing processes that do not require expensive reactors for biomass production and the possibility to scale-up the process in greenhouses. Another advantage is low-cost production, estimated to be 10–50 times lower than products derived from *E. coli* and 140 times lower than production using baculovirus-based insect cells [279]. Tusé and colleagues described a deep analysis of costs of manufacturing in *Nicotiana* host plants of enzymes for diverse applications including a butyrylcholinesterase (rBuChE) for use as a medical countermeasure [280]. Their analyses indicate that cost advantages over alternative platforms can be achieved with plant systems, but also that these advantages depend on the molecule and the relative cost-efficiencies of alternative sources of the same product. Estimations for rBuChE indicate that a dose of 400 mg could be obtained by approximately $234 if an existing toll-manufacturing facility were available, a number significantly below the costs obtainable with blood-extraction processes and substantially lower than those for transgenic approaches [281]. Additional reports estimate that recombinant proteins could be produced in plants at 2–10% of the cost of microbial fermentation systems and at 0.1% of the cost of mammalian cell cultures [282,283]. A great comparison of the cost, applicability, production time, scalability and regulatory compliance of different plant-based platforms is described by Xu and colleagues [268]. Compared with the other plant-based platforms, plant cells are more suitable to the pharmaceutical industry with fewer regulatory and environmental concerns [284]. On the other hand, stable transgenic plants face the major regulatory drawbacks mainly related to GMO environmental concerns. There are also different GMP concerns for products produced from whole plants. Contained, sterile environment used for plant cells in bioreactors meet the same GMP criteria of cell-based platforms, but significant changes are required to adapt for proteins produced in whole-plant systems [285].

Licensed product for human health manufactured in plant-based systems are biologics, as is the case of Elelyso^®^ (Pfizer, New York, USA), a therapeutic recombinant human glucocerebrosidase enzyme taliglucerase alfa produced in carrot-cell suspension culture that was approved by the FDA in 2014 to treat Gaucher disease [286,287,288]. One of the most promising platforms for vaccine development is Proficia™, a proprietary technology of Medicago that uses *Nicotiana benthamiana* plants as manufacturing platform. A phase 3 clinical study using a plant-derived VLP quadrivalent influenza vaccine was recently completed, reporting that the vaccine candidate can provide substantial protection against influenza viruses in adults [289]. More recently, Medicago and GSK announced positive interim phase 2 results for adjuvanted plant-derived VLP COVID-19 vaccine candidate while phase 3 clinical study is ongoing (NCT04636697). Regarding veterinary vaccines, Dow Agro Sciences (Indianapolis, Indiana, USA) obtained United States Department of Agriculture (USDA) approval in 2006 for a plant-cell-culture-based vaccine for poultry against Newcastle disease virus. The vaccine was composed of recombinant hemagglutinin–neuraminidase protein expressed in transgenic tobacco suspension cells [290]. Although the company finally did not commercialize the product, USDA approval defined a landmark success in plant-derived vaccine development. Moreover, many other recombinant viral proteins for human or veterinary diseases produced in plant systems have been tested, including human immunodeficiency virus, Ebola, rotavirus, Japanese encephalitis, foot-and-mouth disease virus and bovine viral diarrhea virus [291,292].

Owing to the fact that plants are edible, they could also serve as delivery vehicle for oral vaccination, leading to reduced productions costs as purification steps could be avoided. This system also offers advantages in terms of storage due to the high stability of the expressed antigens bio-encapsulated within the cell wall of plant cells. Standardization of antigen dose is a key aspect because the concentration of antigen in different parts of plant could not be the same making difficult to standardize vaccine dose [292]. Furthermore, another important challenge is the lack of a proper dosing strategy, because it has been reported that low doses may not be able to induce a sufficient immune response, while high doses may lead to immune tolerance [34].

### 6.6. Microalgae

Microalgae are also of great interest as alternative systems for production of biopharmaceuticals. The species explored thus far include photosynthetic microalgae such as *Chlamydomonas reinhardtii* (*C. reinhardtii*), *Phaeodactylum tricornutum*, *Dunaliella salina* (*D. salina*) and *Chlorella vulgaris* and non-photosynthetic microalgae such as *Schizochytrium* sp. [293]. They show particular advantages, including rapid transformation, high growth rate, ease of cultivation and the ability to perform post-transcriptional modifications and properly fold complex proteins [294]. Microalgae can be cultivated in very low-cost culture media, especially in the case of photosynthetic algae that have minimal nutritional requirements. It is expected that the production of algae-derived biopharmaceuticals will represent similar savings to those estimated for plant-based platforms when comparing to microbial fermentation or mammalian cell cultures [293]. This system also offers high safety profile since they lack toxic endogenous compounds and they do not have risk of animal pathogens contamination. However, some disadvantages of this system include low expression levels and improper glycosylation of proteins [295]. Production in chloroplasts can be successfully performed, but the use of nucleus-based expression, which offers the production of more complex proteins, has yet to be optimized to reach high yields. Current yields in chloroplast expression are typically in the 0.02–2% of total soluble protein range (∼0.03–3 mg/L of culture medium) [293]. *C. reinhardtii* is the most widely used microalgae and several molecular genetic tools are available for this species, including a fully sequenced genome, methods for transformation and mutagenesis, and vectors for secreted or non-secreted recombinant protein production [159]. Vaccine candidates expressed in microalgae are also of great interest for oral administration and numerous reports have identified algae-derived compounds as immunomodulatory molecules. In addition, the algae cell wall serves as bioencapsulation, preventing antigen degradation by enzymes of the gastrointestinal tract [296]. In regard to regulatory aspects, it is also of importance that microalgae as *C. reinhardtii* are accepted as GRAS by the FDA. Current candidates against human diseases expressed in microalgae include vaccines candidates produced in *C. reinhardtii* against malaria [297] or human papillomavirus and vaccine candidates produced in *Schizochytrium* sp. against influenza [298] or Zika virus [299]. Regarding veterinary use, a vaccine candidate produced in *D. salina* against the white spot syndrome virus was reported to significantly reduce the mortality of orally immunized crayfish [300]. A great summary of microalgae characteristics and biopharmaceuticals produced against human and animal diseases is discussed by Rosales Mendoza and colleagues [293].

## 7. Conclusions

Vaccination has been irrefutability useful in the reduction and control of infectious diseases and is still one of the most important strategies to prevent millions of deaths worldwide. All through history, traditional vaccines based on live-attenuated or inactivated pathogens have allowed for the successful prevention of several diseases, even the eradication of smallpox caused by variola virus (VARV) and rinderpest caused by the rinderpest virus (RPV). Nevertheless, they pose drawbacks, and besides safety concerns related to the potential to revert to pathogenic forms, they possess limitations in regard to manufacturing capabilities. Although production processes are well-defined on the basis of previous developments, the production of large volumes of pathogens are required, leading to lengthy manufacturing procedures and a significant lag time between vaccine production and delivery. Production capability also meets limitations when a high number of doses are required, as in the recent COVID-19 pandemic. Construction and commission of a vaccine manufacturing facility is so costly and lengthy, that when peaks of production are required, capabilities are dependent on the limited existing facilities. Furthermore, one of the biggest weaknesses of traditional vaccines is that not all infectious pathogens can be easily cultured, encountering difficulties to address multiple infectious diseases. This problem was partially solved with the emergence of recombinant subunit vaccines. Advances in antigen design and the understanding of their interaction with the immune system in combination with the establishment of recombinant systems for protein expression opened the door to the development of new vaccine candidates for infectious diseases that still had no treatment. Moreover, although subunit vaccines tend to reduce immunogenicity when compared to live vaccines, several approaches are being explored to enhance their efficacy. The great potential of recombinant vaccines resides in the ability to rationally design antigenic sequences. Availability of multiple genome sequences, in addition to bioinformatics tools that enable prediction of antigenic regions and protein structure, allows for the in silico design of sequences for vaccine candidates. What is more, nanotechnology and nanoparticle design for antigen display are leading technologies in recent advances for recombinant vaccine development. One of the main limitations of current vaccine-manufacturing technologies is that they are restricted to producing only one vaccine or a very narrow range of vaccines. Thus, individual manufacturing processes need to be developed for each vaccine, making vaccine development costly, time-consuming and restricted. Platform technologies allow for the standardization of upstream and downstream processes, given that the platform base remains unchanged. Although certain processes would require optimization, these systems provide flexibility, the possibility for multi-product facilities and rapid responses against emerging challenges, such as pandemic emergencies. Considering protein-based vaccines, antigen display systems are potential candidates as vaccine platforms. They permit standardization of manufacturing processes by using a versatile platform that could react fast against emerging necessities. The route of administration of vaccines is also a pivotal aspect, not just for improvement of immunity, but also to solve the bottlenecks of demanding resources for the massive administration of injectable vaccines. We can easily perceive the positive impact that orally administered COVID-19 vaccines would have, as they would not require trained personnel for their administration. Platforms for heterologous protein production are also key aspect in vaccine development. Some of the selection criteria for protein expression platforms are cost and time for development and production processes, and molecular requirements of antigens (disulfide bonds, glycosylation, secretion, assembly of protein complexes, etc.). In general terms, the more complex the antigen to be expressed, the more sophisticated the platform required.

Traditional platforms for recombinant expression such as bacteria, yeast or insect cells have permitted the production of the currently available subunit vaccines for human and animal use. Bacterial expression systems, particularly the one based on the dominant-strain *E. coli*, offer advantages, such as high speed of production and high yields of expression at reduced costs, but they also have limitations in the expression of complex proteins, because post-transcriptional eukaryotic modifications cannot be performed. Although the hepatitis E vaccine for human use and canine leishmaniasis or feline leukemia veterinary vaccines are produced in this system, the number of products in the market is still limited, most likely due to the fact that complex proteins are usually required as vaccine candidates. Thus, yeast and insect cells are the preferred platforms form human vaccine production and for expression of assembled antigens, such as VLPs. Concerning animal health, the insect cell is notably the dominant platform for recombinant vaccine manufacturing. Although a great number of biopharmaceuticals, such as therapeutic antibodies, are currently produced in mammalian cells, the number of marketed vaccines is still limited due to the higher costs and requirements associated with development and manufacture with this system. Nonetheless, a human vaccine against herpes zoster virus produced in CHO cells was recently approved for commercialization by EMA and FDA.

Alternative production platforms that can overcome all the mentioned limitations in vaccine production are being increasingly demanded. Bacteria and yeast are being engineered to decrease cost and time of production and to improve expression yields and complexity of heterologous proteins. Pressing demands of cost-effective platforms for vaccine production have led to the emergence of alternative systems, such as insects or plants. These platforms circumvent the use of expensive bioreactors and culture media, minimizing manufacturing requirements. One of the main disadvantages of the plant system is the long period involved with the establishment of transgenic plants. Furthermore, high yield expression of complex proteins is still challenging. Whole plants and plant cells have been widely explored for expression of therapeutic proteins, and a therapeutic recombinant human glucocerebrosidase enzyme produced in carrot cells is already commercialized. Insect pupae and larvae are emerging as promising systems for protein production due to their capability to express complex proteins with high yields. The use of insects as single bioreactors offers versatility and easy scale-up, simplifying the requirements to construct and equip a vaccine-manufacturing facility. Although just two biologics produced in insect larvae are marketed at the moment, several vaccines candidates are under development showing promising results as an alternative platform for vaccine development. As a final point, the algae expression system has also appeared as a promising platform for cost-effective production of proteins, due to the high growth rate and low requirements; however, optimization of nuclear expression is still required to achieve high-yield expression of complex proteins. The final suitability of the abovementioned platforms will depend on the nature of the vaccine candidates. Although these platforms and technologies have emerged as promising alternatives to vaccine production, further effort is required, especially regarding the reduction of costs and time associated with vaccine development. The recent COVID-19 pandemic has revealed the importance of quick responses to vaccine manufacturing, as well as that the time required for vaccine development and delivery is critical. To achieve this goal, flexible and versatile vaccine-manufacturing platforms are essential to overcome current and future vaccination demands.

## Figures and Tables

**Table 1:** Recombinant protein vaccines approved for human use.

Production System	Host	Disease	Vaccine Name (Manufacturer)	Regulatory Approval	Antigen	Vaccine Type	Reference
Bacteria	*E. coli*	HEV	Hecolin^®^ (Innovax)	2012 *	ORF2 HEV	VLP	[89,90]
Yeast	*S. cerevisiae*	HBV	Recombivax HB^®^ (Merck & Co.)	1986 FDA	HBsAg	VLP	[91,92]
	*S. cerevisiae*	HBV	Engerix^®^-B (GSK)	1989 FDA	HBsAg	VLP	[93,94]
	*S. cerevisiae*	HBV	HBvaxPRO^®^ (Merck & Co.)	2001 EMA	HBsAg	VLP	[95]
	*S. cerevisiae*	HBV	Fendrix^®^ (GSK)	2005 EMA	HBsAg	VLP	[96]
	*H. polymorpha*	HBV	Heplisav-B^®^ (Dynavax)	2017 FDA 2021 EMA	HBsAg	VLP	[97,98]
	*S. cerevisiae*	HPV	Gardasil^®^ (Merck & Co.)	2006 FDA 2006 EMA	L1 HPV 6, 11, 16, 18	VLP	[99,100,101]
	*S. cerevisiae*	HPV	Gardasil-9^®^ (Merck & Co.)	2014 FDA 2015 EMA	L1 HPV 6, 11, 16, 18, 31, 33, 45, 52, 58	VLP	[102,103]
	*S. cerevisiae*	Malaria	Mosquirix^®^ (GSK)	2015 EMA (outside EU)	RTS,S	VLP	[104,105,106]
Insect cells	High Five™	HPV	Cervarix^®^ (GSK)	2007 EMA 2009 FDA	L1 HPV 16, 18	VLP	[107,108,109]
	ExpresSF+^®^	Influenza	FluBlok^®^ (Sanofi Pasteur)	2013 FDA	HA trivalent	Subunit	[110,111]
	ExpresSF+^®^	Influenza	Flublok Quadrivalent^®^/Supemtek^®^ (Sanofi Pasteur)	2016 FDA 2020 EMA	HA quadrivalent	Subunit	[112]
Mammalian cells	CHO	Herpes zoster	Shingrix^®^ (GSK)	2017 FDA 2018 EMA	gE	Subunit	[113,114,115]

* Granted a marketing authorization in China by China Food and Drug Administration. Phase 1 clinical trial approved by FDA (NCT03827395). HEV: Hepatitis E virus; HBV, hepatitis B virus; HBsAg, hepatitis B surface antigen; VLP, virus-like particle; HPV, human papillomavirus; RTS,S, *P. falciparum* protein fused with hepatitis B surface antigen (RTS) combined with hepatitis B surface antigen (S); HA, hemagglutinin; CHO, Chinese hamster ovary cell; gE, herpes zoster virus glycoprotein E.

**Table 2:** Recombinant protein vaccines approved for veterinary use.

Production System	Host	Disease	Vaccine Name (Manufacturer)	Regulatory Approval	Antigen	Vaccine Type	Reference
Bacteria	*E. coli*	FeLV	Leucogen^®^ (Virbac)	2009 EMA	p45 FeLV-envelope antigen	Subunit	[116,117]
	*E. coli*	Canine leishmaniasis	Letifend^®^ (LETI Pharma)	2016 EMA	*L. infantum* MON-1 Q protein	Subunit	[118]
Insect cells	Sf21	CSF	Porcilis Pesti^®^ (Merck & Co.)	2000 EMA	E2 glycoprotein	Subunit	[119,120]
	Sf21	CSF	Bayovac^®^ CSF E2 * (Bayer)	2001 EMA	E2 glycoprotein	Subunit	[119,121]
	Sf+	PCV2	Ingelvac CircoFLEX^®^ (B. Ingelheim)	2006 USDA 2008 EMA	ORF2 protein PCV2a	VLP	[122,123,124]
	Sf9	PCV2	Circumvent^®^ PCV (Merck & Co.)	2007 USDA	ORF2 protein PCV2a	VLP	[125]
	Sf21	PCV2	Porcilis^®^ PCV (Merck & Co.)	2009 EMA	ORF2 protein PCV2a	VLP	[126]
	n.s.	PCV2	CircoGard^®^ (Pharmgate)	2017 USDA	ORF2 protein PCV2b	VLP	[127]
	n.s.	PPV	ReproCyc^®^ ParvoFLEX (B. Ingelheim)	2019 EMA	PPV 27a VP2	VLP	[128]

* Bayovac^®^ CSF E2 vaccine has been discontinued. FeLV, feline leukemia virus; CSF, classical swine fever; PCV2, porcine circovirus type 2; PPV, porcine parvovirus. Published documents for CircoGard^®^ and ParvoFLEX^®^ do not specify the insect cell line used for vaccine production (n.s.).

## Data Availability

Not applicable.
